# Investigation on Elastic Constants of Microfibril Reinforced Poly Vinyl Chloride Composites Using Impulsive Excitation of Vibration

**DOI:** 10.3390/polym14235083

**Published:** 2022-11-23

**Authors:** Sampath Aravindh, Gopalan Venkatachalam

**Affiliations:** 1 School of Mechanical Engineering, Vellore Institute of Technology, Chennai 600127, India; 2 Centre for Innovation and Product Development, Vellore Institute of Technology, Chennai 600127, India

**Keywords:** coir fiber powder, chemical treatments, P-polyvinylchloride, injection moulding, Box–Behnken design, impulse excitation of vibration

## Abstract

The creation of tenable green composites is in high demand, due to ecologically available resources paving the way for applications to thrive in the manufacturing, aerospace, structural, and maritime industries. Hence, it is vital to understand the performance characteristics of natural fiber-reinforced polymer composites. The elastic constants of coir fiber powder-reinforced plasticized polyvinyl chloride composite are determined using impulsive excitation vibration in this study. The optimization study on the elastic constants was carried out using Box–Behnken experimental design, based on response surface methodology, having three factors of fiber content (wt.%), fiber size (μm) and chemical treatments. The results were evaluated using analysis of variance and regression analysis. Additionally, experimental and optimized results were compared, leading to error analysis. Young’s modulus of 18.2 MPa and shear modulus of 6.6 MPa were obtained for a combination of fiber content (2 wt%), fiber size (225 μm), and triethoxy (ethyl) silane treatment, which is suitable for various electrical, automotive, etc., applications.

## 1. Introduction

Natural fiber-reinforced polymer composites (NFPCs) have become a viable replacement for synthetic fiber-reinforced composites [1] in recent decades due to their low cost, environmental friendliness [2], light weight, and mechanical qualities [3]. Although natural fiber composite products are being produced and marketed, the majority of the technologies are still at the stages of research and development (R&D) [4]. Natural fiber composites (NFCs) are employed in a variety of automotive applications, including rear view mirrors, two-wheeler visors [5], door panels and head rests [6] as well as structural applications such as indoor element housings [7]. Among natural fibers, coir fibers are considered because of their high availability, high elongation at break, high lignin content, low density and low cost [8,9,10] properties.

Because of their better recyclability, increased toughness and ability to produce rapid manufacturing processes, natural fibers as reinforcing agents in thermoplastic polymer composites have gained increasing applications in the automotive, aerospace, electrical and electronics, industrial, and medical fields [11,12]. Plasticized polyvinyl chloride (PVC), one of the thermoplastics, is predominately used for its inexpensive, durable and flexible [13] properties. Various applications include construction, domestic, transportation packaging, medical, and clothing and PVC is further used in conveyer belts, inflatable and sports materials [14]. Natural fiber reinforced with plasticized polyvinyl chloride (PVC) composites has satisfactory mechanical properties and is more environmentally friendly than pure polyvinyl chloride (PVC) [15,16].

The main drawbacks of natural fiber-reinforced polymer composite are higher water absorptiveness, inferior fire resistance and lower mechanical properties [17,18]. Chemical treatments such as polymer coupling agents, silane, alkali and isocyanate treatments, as well as permanganate [19,20], are utilised to increase interfacial adhesion between the natural fiber surface and polymer [21,22,23]. Sodium hydroxide (NaOH) treatment with a 3 h immersion stage resulted in the maximum tensile strength [24]. Coir fiber composite, treated with 5% (*w*/*v*) potassium hydroxide (KOH), offers better mechanical qualities than untreated coir fiber composite [25]. The flexural and tensile strengths of methacryloxy-propyltrimethoxy (silane treatment) bamboo fibers are 36.1 MPa and 54.7 MPa, respectively, which are 15.4% and 23.6% higher than untreated bamboo fibers-reinforced polypropylene composites [26].

The impulsive excitation of vibration is a vibration-based approach and non-destructive technique which uses material resonance frequencies to evaluate elastic properties [27]. The elastic characteristics from non-destructive techniques are in high-quality conformity with the results from three-points bending and shearing methods [28]. In free vibration characteristics, natural frequency increases with the addition of glass in sandwich composites such as sisal/PVC/glass and banana/PVC/glass [29].

The natural frequency analysis for different composites indicates different natural frequencies such as HDPE and bamboo fiber composites with 43.6 Hz, ABS and bamboo fiber composites with 40.23 Hz and HDPE, ABS and bamboo fiber composites together indicates 43.065 Hz. This exhibits the innate ability of HDPE to remain strong even under intense loading [30]. The elastic constant of jute fiber-reinforced composites such as Young’s modulus (6.8 GPa), shear modulus (2.5 GPa) and Poisson’s ratio (0.36) are determined by the impulsive excitation of vibration [31]. The natural frequency of composite material depends on factors including geometry, mass, dimensions [32].

Fabrication of composites involves different conventional manufacturing processes such as extrusion and injection moulding [33]. Further, they are used in the direct integration of short fibers into polymers [34,35]. In the midst of conventional methods, injection moulding increases the spreading of fibers to resin and improving the mechanical characteristics of the composites [36,37].

Response surface methodology (RSM), introduced by Box and Wilson [38], is used to optimize the conditions for the manufacturing of composites [39,40]. Central composite design and Box–Behnken design are the two most prevalent designs utilised in response surface methodology (RSM) [41,42]. Among response surface methodology, Box–Behnken design is better because it does not have axial points, thus all design points fall within the safe [43,44]. ANOVA can be used to decide the impact of each input factor on the response variable. Thus, response values are entered into statistical software such as Design Expert, Minitab [45,46] in order to execute analysis of variance (ANOVA) and to create a regression equation using response variables [47,48].

From the literature review, it is concluded that the composite material with a combination of P-PVC and coir fiber powder has not been reported using statistical techniques. Moreover, investigation of the elastic constant of such composite material is not attempted for the above combination.

As a result, a novel study is carried out where the Box–Behnken design (BBD) is utilized to examine the elastic constants (Young’s modulus (E), shear modulus (G) and Poisson’s ratio) of coir fiber-reinforced plasticized polyvinyl chloride composites in order to obtain optimized parameters of interest. The effect of independent variables (fiber content (wt.%), fiber size (μm), and chemical treatments) on the dependent variables (elastic constants) are studied to find maximum Young’s and shear moduli.

## 2. Materials and Methodology

### 2.1. Raw Materials

Raw coir fiber was procured from provisioner (Go Green product, Chennai, India) and pulverised to various micron sizes for use as reinforcing filler. The chemicals used for treatments for the uprooting of cellulose microfibrils were triethoxy (ethyl) silane, sodium hydroxide (NaOH), potassium hydroxide (KOH), acetic acid and demineralised water. These chemicals were procured from SRL and NICE chemicals. Plasticized polyvinyl chloride was procured from (Sigma Aldrich, Bangalore, India) and used as the polymer matrix.

### 2.2. Experimental Design by Response Surface Methodology (RSM)

In this paper, Box–Behnken design (BBD) of RSM is used, which comprises three-level, three-factor and requires 15 runs. The three-levels of factorial design must be equally spaced values, such as −1, 0 and +1. The three factors selected were fiber content (2 wt.%, 4 wt.% and 6 wt.%), fiber size (75 μm, 150 μm and 225 μm) and chemical treatments ((1). (triethoxy (ethyl) silane), (2). (sodium hydroxide) and (3). (potassium hydroxide)) as indicated in Table 1. The generalised second-order polynomial model is utilised in the response surface approach, as shown in Equation (1). As stated in Table 2, there are 15 experimental points, 12 of which are factorial design points and 3 of which are replicas of centre points. Minitab statistical software version 19 was used to apply the Box–Behnken design (BBD) to the experimental data.

(1) $$Y=b_{0}+\sum_{i=1}^{3}b_{i}X_{i}+\sum_{i=1}^{3}b_{ii}X_{i}{}^{2}+\sum_{i=1}^{2}\sum_{j>1}^{3}b_{ij}X_{i}X_{j}+\ldots$$

where

*Y* is a response variable

*b*_0_ is a constant value.

*bi* is a linear coefficients

*b_ii_* is a coefficient of a quadratic equation.

*bij* is a coefficient of interaction.

*Xi* denotes a dimensionless coded independent variable

**Table 1:** Levels and codes preferred for Box–Behnken design.

Factor	Variables	Coded Levels of Variables		
		−1	0	+1
A	Fiber content (wt.%)	A1 (2)	A2 (4)	A3 (6)
B	Fiber size (μm)	B1(75)	B2(150)	B3(225)
C	Chemical treatments	C1 (1)	C2 (2)	C3 (3)

**Table 2:** Box–Behnken design with coded/actual values for three-factors of factorial design.

Run No.	Variables (Coded Values)			Variables (Actual Values)		
	Fiber Content (wt.%)	Fiber Size (μm)	Chemical Treatments	Fiber Content (wt.%)	Fiber Size (μm)	Chemical Treatments
1	+1	0	+1	6	150	3
2	−1	0	−1	2	150	1
3	0	0	0	4	150	2
4	−1	−1	0	2	75	2
5	+1	+1	0	6	225	2
6	0	+1	−1	4	225	1
7	0	+1	+1	4	225	3
8	0	0	0	4	150	2
9	0	−1	−1	4	75	1
10	0	−1	+1	4	75	3
11	−1	0	+1	2	150	3
12	+1	0	−1	6	150	1
13	0	0	0	4	150	2
14	+1	−1	0	6	75	2
15	−1	+1	0	2	225	2

### 2.3. Chemical Treatment for Fibers

**Figure 1:**
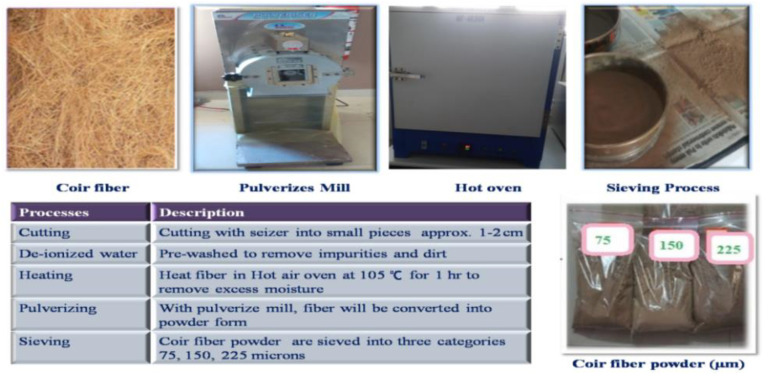
Pulverization process.

To remove impurities and dirt, raw coir fibers are trimmed to 1–2 cm length and pre-washed with de-ionized water. The fibers are then dried for 1 h in a hot air oven at 105 °C to eliminate excess moisture. Coir fibers are fully dried and ground using a pulverising method (Saral Pulverizer, Gujarat, India), followed by size severance using a sieve shaker [49] as shown in Figure 1.

**Figure 2:**
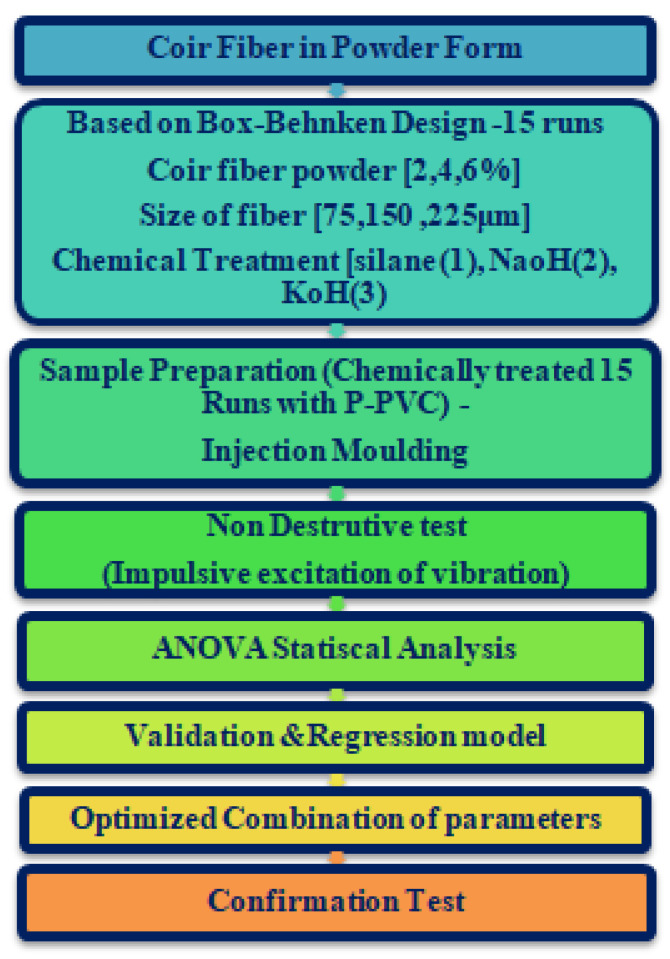
Flowchart for the sample preparation using impulsive excitation of vibration.

**Figure 3:**
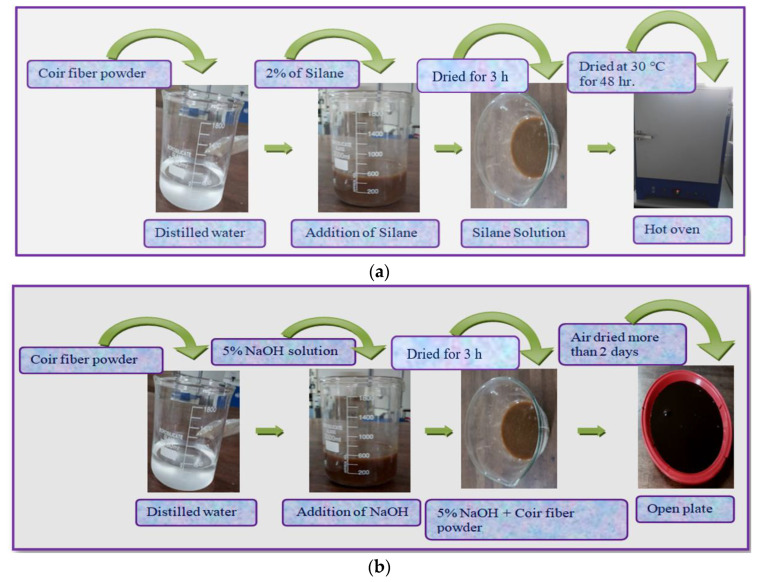

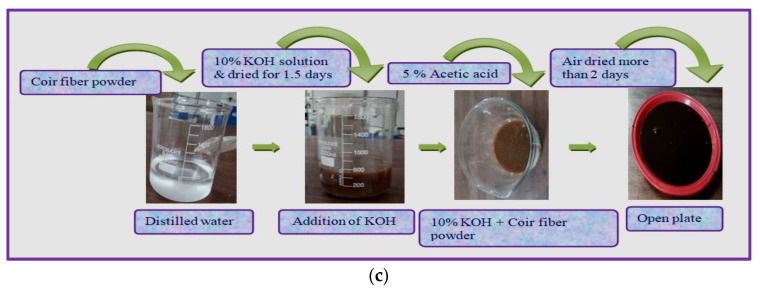
(**a**) Triethoxy(ethyl) silane treatment [50]. (**b**) Sodium hydroxide (NaOH) treatment [51]. (**c**) Potassium hydroxide (KOH) treatment [52].

To prepare coir fiber reinforced with plasticized polyvinyl chloride composite in accordance with the BBD, which is depicted in Figure 2, the coir fiber powder content (2 wt.%, 4 wt.%, and 6 wt.%) in combination with the size of the fiber (75 μm, 150 μm, and 225 μm) are chemically treated with triethoxy(ethyl)silane (1), sodium hydroxide (NaOH) (2) and potassium hydroxide(KOH) (3). Figure 3 describes the procedure for chemical treatments.

### 2.4. Injection Moulding Process

**Figure 4:**
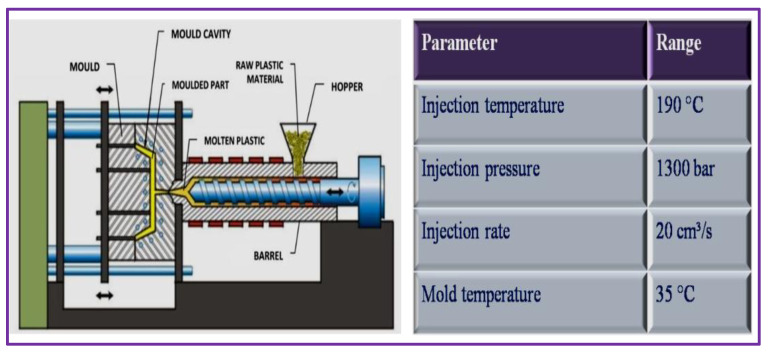
Digital Injection Moulding Machine.

Samples were prepared by reinforcing coir fiber powder with plasticized-PVC according to BBD, as presented in Table 2. A hydraulic injection moulding machine was used for the manufacturing of 15 samples. By shearing action of the screw, coir fiber powder and plasticized PVC were fed into a barrel from a hopper, in which the materials were melted. Then the screw stopped rotating and materials moved forward into the mould. The cooling cycle began once the mould was entirely filled and the screw continued to apply consistent pressure on the polymer–fiber mixture. The mould was opened when the cycle finished and a sample with dimensions of 115 mm × 95 mm × 3 mm was expelled, as illustrated in Figure 4.

### 2.5. Impulsive Excitation of Vibration

**Figure 5:**
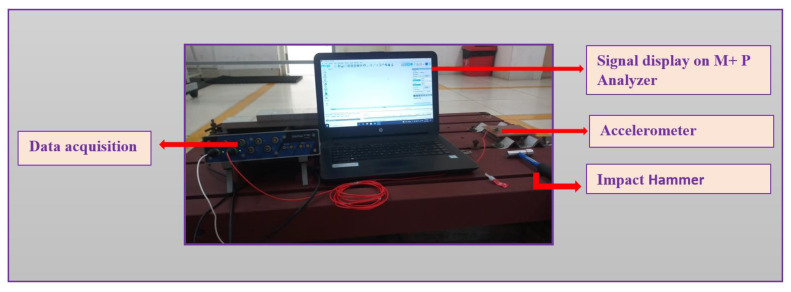
Sample setup for impulsive excitation of vibration.

The analysis of the elastic constant of materials using impulse excitation of vibration is a non-destructive method. It is non-destructive to material qualities and can assess mechanical properties including resonance frequency and internal friction. As per ASTM 1876 [53], the elastic constants (Young’s modulus (E) and shear modulus (G)) are computed from resonance frequency under various conditions such as torsion mode, bending mode or bending-torsion mode. An impact hammer is used to excite the sample in bending or torsion mode. The vibration caused by the collision on the surface of the samples is detected using an accelerometer. Data acquisition is used to gather the signals, which are transferred to M + P analyzer software to perform Fourier transform (FFT) analysis, as shown in Figure 5.

By using this technique, elastic constants are computed. Equations (2) and (3) are used to calculate elastic constant values from resonance frequency measurements under bending and torsion circumstances:

(2) $$E=0.9465\;\left(\frac{{\mathrm{mf}}_{f}^{2}}{b}\right)\left(\frac{L^{3}}{t^{3}}\right)\;T$$

(3) $$G=\frac{4{\mathrm{Lmf}}_{t}^{2}}{\mathrm{bt}}\left(\frac{B}{1+A}\right)$$

where E is the Young’s modulus (MPa), G is the shear modulus (MPa), L is the length of bar (mm), m is the mass (g), t is the thickness of the bar (mm), b is the width of the bar (mm), f_f_ is the flexural frequency (Hz), f_t_ is the torsion frequency (Hz), T, A, and B are the resonance frequency correction coefficients (Hz). Equations (4)–(6) illustrate B, A and T, respectively.

(4) $$B=\frac{b/t+\;t/b}{4\left(t/b\right)-2.52{\left(t/b\right)}^{2}+0.21{\left(t/b\right)}^{6}}$$

(5) $$A=\left[\frac{\left[0.5062-0.8776\left(b/t\right)\right]+0.3504{\left(b/t\right)}^{2}-0.0078{\left(b/t\right)}^{3}\;}{\left[12.03\left(b/t\right)+9.892{\left(b/t\right)}^{2}\right]}\right]\;$$

(6) $$T=1.000+6.585{\left(t/L\right)}^{2}$$

Poisson’s ratio is determined using data from Young’s modulus (E) and shear modulus (G). Thus Equation (7) is stated as follows.

(7) μ=(E/2G)−1

### 2.6. Data Analysis

The Minitab 19 statistical software is used to evaluate the experimental data produced by impulsive excitation of vibration. The methodology for evaluating the elastic constants results (Young’s modulus, shear modulus) is as follows: performing an analysis of variance (ANOVA), generating a regression equation through response variables, a Pareto chart of standardized effects, a response 3D surface plot and a main effect plot. Thus, dependent and independent variables are examined to determine the optimum combination for elastic constants.

## 3. Result and Discussion

### 3.1. Morphology of Microfibrils

**Figure 6:**
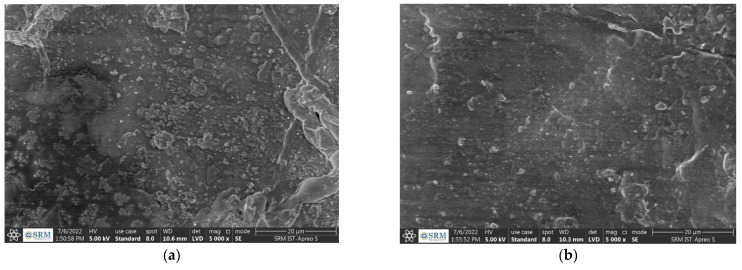
SEM micrographs of coir fiber-reinforced P-PVC. (**a**) 6 wt.% of fiber + 150 μm + triethoxy (ethyl) silane (**b**) 2 wt.% of fiber + 75 μm+ NaOH.

Figure 6 depicts the surface topography and composition of the sample under scanning electron microscope (SEM) [54] of the coir fiber-reinforced P-PVC composites. SEM images are seized to 20 μm to view the presence of coir fiber powder in composite materials. It is evident that Coir fiber powder (In the form of white color structure) is reinforced with P-PVC in Figure 6.

### 3.2. Analysis of Impulse Excitation of Vibration

**Figure 7:**
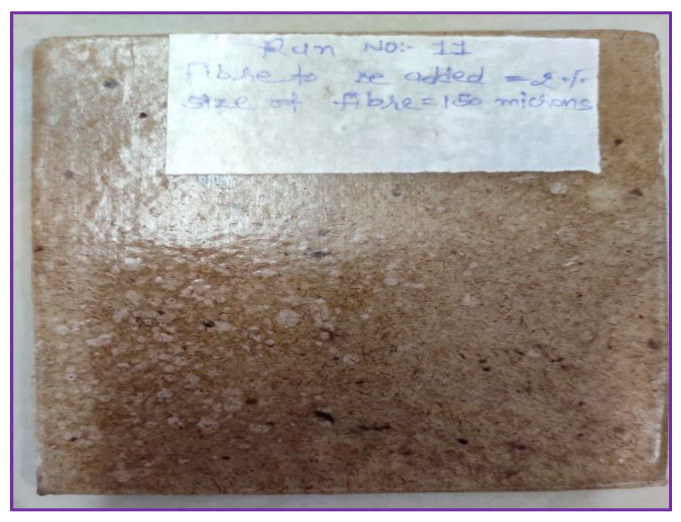
Specimen of composites.

**Figure 8:**
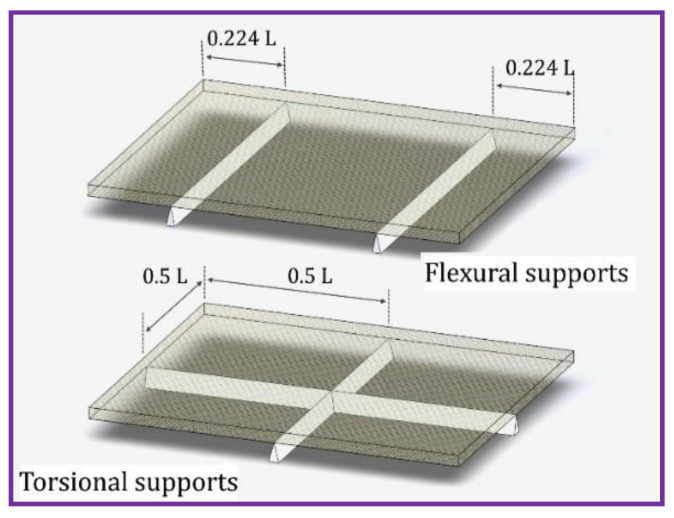
Support position for locating a sample.

The specimens were prepared according to ASTM1876, pertaining to impulse excitation of vibration, as shown in Figure 7. In an effort to attain the resonance frequency of the sample using flexural mode, the sample is positioned with two support systems located at 0.224 mm distance from the total length of the specimen at each end. To acquire the resonance frequency of the sample using torsional mode, the specimen is located at the centre point of the symmetrical and cross-shaped support as illustrated in Figure 8.

The elastic constants of coir fiber-reinforced P-PVC composites, calculated using the determined frequencies from the impulse excitation of the vibration, are provided in Table 3.

From the experiment, a high Young’s modulus of 17.2 MPa was obtained for the combination of 2 wt.% fiber content, 150 μm fiber size and triethoxy(ethyl)silane treatment and low of 8.6 MPa for the combination of 6 wt.% fiber content, 225 μm fiber size and sodium hydroxide treatment. The highest shear modulus of 6.2 MPa was obtained for the combination of 2 wt.% fiber content, 150 μm fiber size and triethoxy (ethyl) silane treatment and low of 3.1 MPa for the combination of 6% fiber content, 225 μm fiber size and sodium hydroxide treatment. From Young’s modulus (E) and shear modulus (G), Poisson’s ratio was computed and ranged from 0.36 to 0.37.

### 3.3. Model Selection and ANOVA Analysis for Elastic Constants

Table 4 shows the experimental and predicted response for all 15 samples obtained from Minitab software. Residual errors are also calculated between experimental and predicted response.

The results of Young’s modulus and shear modulus for coir fiber reinforced P-PVC Composites are explored further using ANOVA to decide the significant variables. By using simple regression analysis to response, ANOVA quadratic models for the elastic constant (Young’s modulus and shear modulus) of three factors are shown in Equations (8) and (9).

Young’s modulus(MPa) = 20.18 − 1.59 A + 0.0576 B − 8.90 C + 0.026 A×A + 0.000081 B×B + 2.438 C×C − 0.00403 A×B+ 0.808 A×C − 0.03656 B×C(8)

Shear modulus(MPa) = 7.35 − 0.573 A + 0.0210 B − 3.24 C + 0.0078 A×A + 0.000029 B×B + 0.885 C×C − 0.00146 A×B + 0.297 A×C − 0.01332 B×C(9)

where A, B, C are fiber content (%), fiber size (μm) and chemical treatments, respectively. These quadratic equations are utilized to generate Young’s modulus and shear modulus predictions for each variable. For all samples, errors are less than 11%, showing the authenticity of developed regression equations.

The ANOVA for the Young’s modulus (quadratic model) is explained in Table 5. The *p*-value for the model is less than 0.05, demonstrating the statistical significance of the model. In other factors, such as C, C×C, A×C and B×C, *p*-values are less than 0.05. The R^2^ value for the model is 93.21%, emphasizing that the model is significant.

The ANOVA for the shear modulus (quadratic model) is revealed in Table 6. The *p*-value for the model is less than 0.05, representing its statistical significance. In other factors, such as C, C×C, A×C and B×C, *p*-values are less than 0.05. The R^2^ for the model is 93.34%, implying that the model is significant.

## 4. Effect of Parameter for Elastic Constants

### 4.1. Response Surface 3D Interaction

To demonstrate the major and interacting effects of independent and response variables, 3D response surface plots were obtained. These graphs are simple to comprehend and are useful for visually representing numerical data.

**Figure 9:**
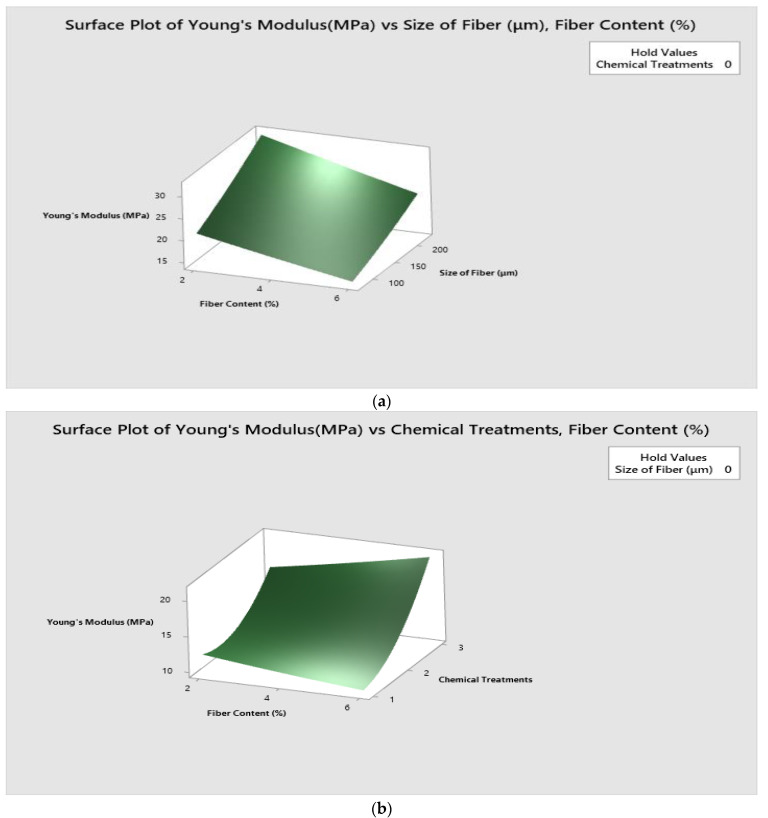

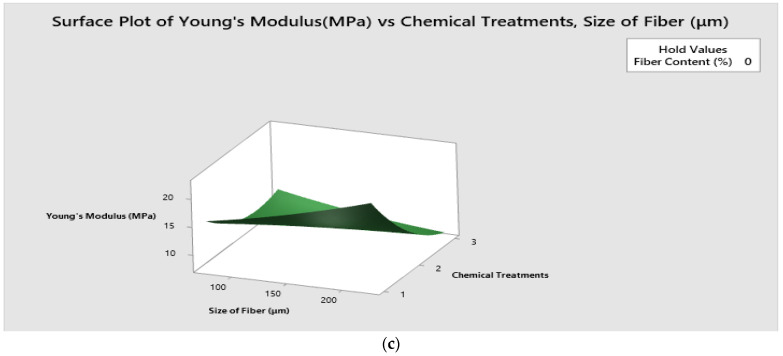
Surface plot for Young’s modulus. (**a**) Surface plot of Young’s modulus (MPa) vs size of fiber (μm), fiber content (wt.%) (**b**) Surface plot of Young’s modulus (MPa) vs chemical treatments/fiber content (wt.%). (**c**) Surface plot of Young’s modulus vs. chemical treatments/fiber size (μm).

Figure 9 shows the surface 3D plot of interaction between input parameters such as fiber content (%), fiber size (μm) and chemical treatments on Young’s modulus.

Figure 9a explains the surface plot of Young’s modulus vs. fiber size (μm)/fiber content (wt.%). It is palpable that high Young’s modulus is observed for the combination of 2% fiber content and 225 μm fiber size.

Figure 9b illustrates the surface plot of Young’s modulus vs. chemical treatments/fiber content (wt.%). It is inferred that high Young’s modulus is observed for the combination of 6% fiber content and potassium hydroxide treatment.

Figure 9c elucidates the surface plot of Young’s modulus vs. chemical treatments/fiber size (μm). It culminated that a high Young’s modulus was observed for the combination of triethoxy (ethyl) silane treatment and 225 μm fiber size.

**Figure 10:**
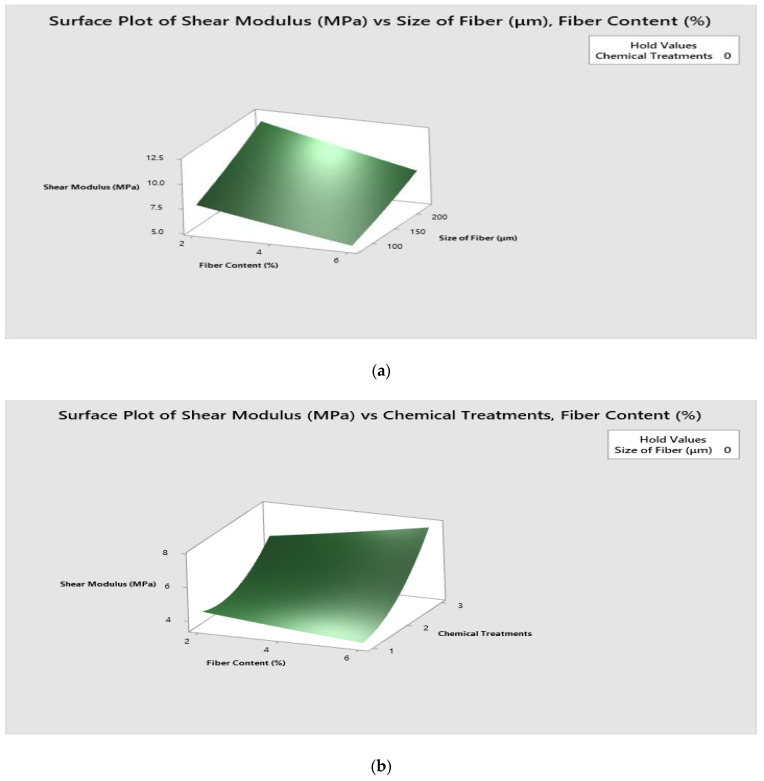

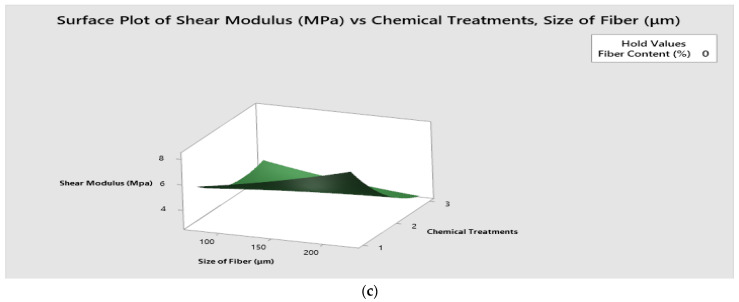
Surface plot for Shear modulus. (**a**) Surface plot of shear modulus vs. fiber size (μm)/fiber content (wt.%). (**b**) Surface plot of shear modulus vs. chemical treatments/fiber content (wt.%). (**c**) Surface plot of shear modulus vs. chemical treatments/fiber size (μm).

Figure 10 shows the surface 3D plot of interaction between input parameters such as fiber content (%), fiber size (μm) and chemical treatments on shear modulus.

Figure 10a explains the surface plot of shear modulus vs. fiber size (μm)/fiber content (wt.%). One can understand from the figure that the combination of 2% fiber content and 215–225 μm fiber size offers high shear modulus.

Figure 10b illustrates the surface plot of shear modulus vs. chemical treatments/fiber content (wt.%). It is visible that high shear modulus is observed for the combination of 6% fiber content and potassium hydroxide treatment.

Figure 10c explicates the surface plot of shear modulus vs. chemical treatments/fiber size (μm). It is theorized that high shear modulus is observed for the combination of triethoxy (ethyl) silane treatment and 225 μm fiber size.

### 4.2. Main Effects Plots

**Figure 11:**
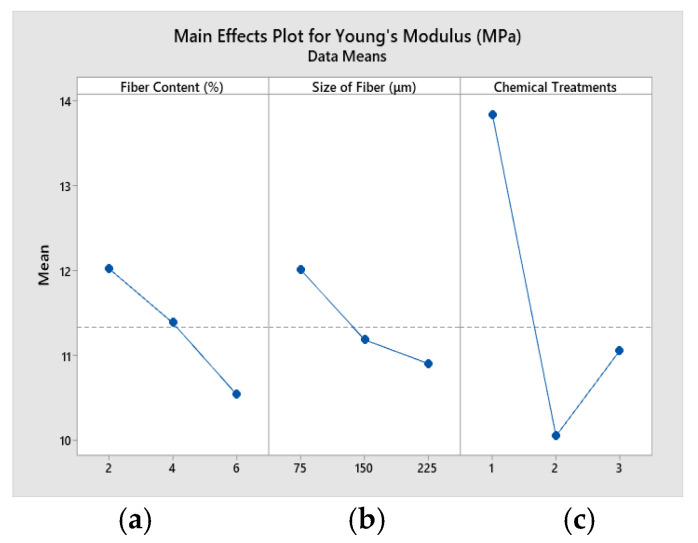
Main effects plot for Young’s modulus. (**a**) fiber content (%) (**b**)size of fiber (**c**) chemical treatments.

Figure 11 shows the main effects plot for Young’s modulus of the composites. Figure 11a shows a rise in Young’s modulus when fiber content is at 2% and Young’s modulus decreases with increase in fiber content from 3% to 6%. Figure 11b exhibits that Young’s modulus increases when fiber size decreases from 75 μm to 225 μm. Figure 11c manifests that Young’s modulus is high when triethoxy (ethyl) silane-treated fiber is used in composites. It also indicates low Young’s modulus when fiber is treated with sodium hydroxide and potassium hydroxide.

**Figure 12:**
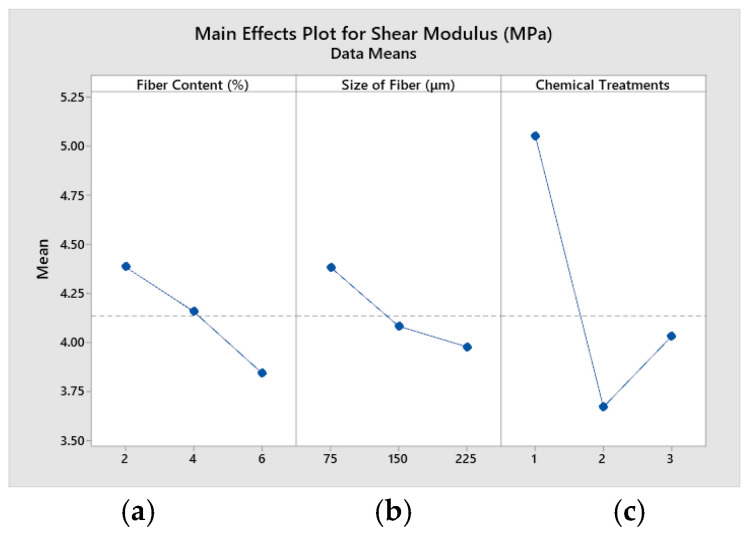
Main effects plot for shear modulus. (**a**) fiber content (%) (**b**) size of fiber (**c**) chemical treatments.

Figure 12 shows the main effects plot for shear modulus of the composites. Figure 12a reveals that shear modulus increases with decrease in fiber content from 2% to 6%. Figure 12b exhibits that shear modulus increases when fiber size decreases from 75 μm to 225 μm. Figure 12c indicates that shear modulus is high when triethoxy (ethyl) silane-treated fiber is used in composites, as compared with other treatments.

When a silanol molecule (Triethoxy(ethyl)silane) interacts with the hydroxyl groups in the cell wall of lignocellulosic materials, silanation takes place. If the components of hydroxyl groups, such as pectin, lignin, and hemicellulose, are successfully removed from the fiber, there is a successful interfacial adhesion between the coir fiber and P-PVC. As a result, the interfaces of the two phases form a powerful chemical bond which tends to increase the Young’s modulus and shear modulus.

**Figure 13:**
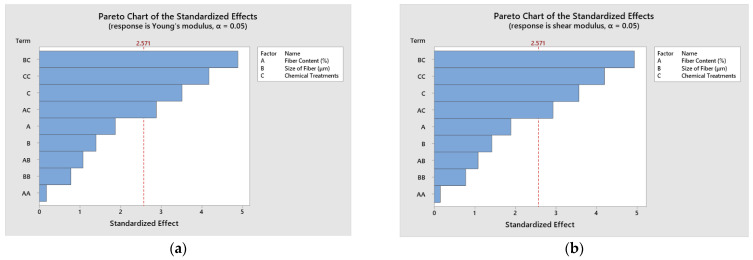
Pareto charts of the standardized effects on elastic constants. (**a**) Young’s modulus (**b**) shear modulus.

Figure 13a,b show the effect of different constituents of the regression equation on the elastic constants. The influence of fiber size and chemical treatments shows maximum Young’s modulus, as illustrated in Figure 13a. The influence of fiber size and chemical treatments shows maximum shear modulus, as exhibited in Figure 13b.

### 4.3. Optimization and Verification of the Model

**Figure 14:**
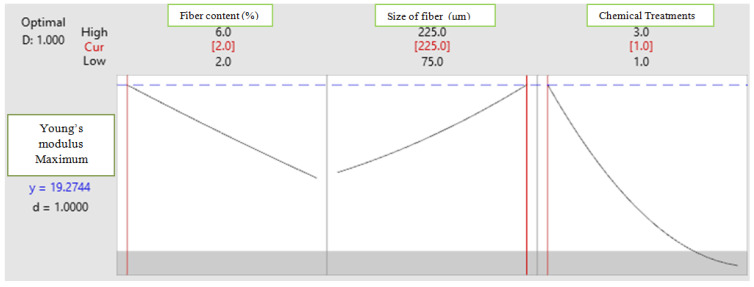
Response optimization plot for Young’s modulus.

**Figure 15:**
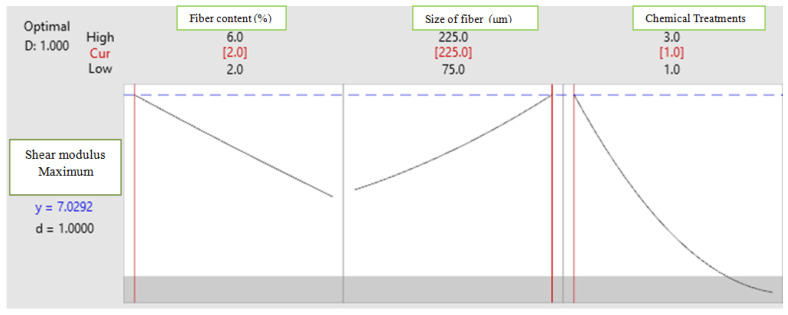
Response optimization plot for shear modulus confirmation test.

To achieve high Young’s modulus and shear modulus, optimum combinations are obtained using the response optimization plot (Figure 14 and Figure 15).

Figure 14 represents a response optimization plot for Young’s modulus, where the X axis shows fiber content (wt.%), fiber size (μm) and chemical treatment and Y axis shows Young’s modulus. It illustrates that a high Young’s modulus is obtained for the combination of coir fiber content with 2 wt%, fiber size with 225 μm and treatment with triethoxy (ethyl) silane.

Figure 15 represents response optimization plot for shear modulus, where the X axis shows fiber content (wt.%), fiber size (μm) and chemical treatment and Y axis shows shear modulus. It illustrates that high shear modulus is obtained for the combination of coir fiber content with 2 wt%, fiber size with 225 μm and treatment with triethoxy (ethyl) silane.

Table 7 explicates the confirmation test for the optimization process, which shows the error analysis for elastic constants between experimental and optimization values. For Young’s modulus, the error is 5.186% which shows validity for optimization process. Similarly, for shear modulus, the error is 5.776% which shows the legitimacy of the optimization process.

## 5. Conclusions

The Box–Behnken design (BBD) approach is utilized to obtain optimum combination of input parameters for high values of responses. Fiber content (wt. %), fiber size (μm), and chemical treatments were the variables studied in this three-level, three-factor investigation. All the specimens were prepared and tested using the impulsive excitation of vibration. Using Minitab software, elastic constants (Young’s modulus and shear modulus) were tabulated, examined and optimised. R^2^ values of 93.21% for Young’s Modulus and 93.34% for shear modulus were obtained by ANOVA. The experimental value and predicted value from regression equation were found to be in good agreement. With the help of 3D response surface graphs and main effects plots, the effects of three factors on Young’s modulus and shear modulus were evidently explained.

By using a response optimizer, a high Young’s modulus value of 19.2 MPa was obtained for the combination of fiber content (2 wt. %), fiber size (225 μm) and triethoxy (ethyl) silane treatment. A high shear modulus value of 7 MPa was gleaned for the combination of fiber content (2 wt. %), fiber size (225 μm) and triethoxy(ethyl)silane treatment. A confirmation test was performed to validate the optimized results and the error was found to be less than 6%. From optimization, it is understood that these combinations tender high Young’s and shear moduli. The addition of coir fiber increases green content in the composite. The addition of coir fiber promotes bio-degradability/recyclability of the composite. This work encourages industries, such as automotive, electrical, etc., to utilize the developed composite as eco-friendly composite material.

## Figures and Tables

**Table 3:** Resonance frequency and elastic constants of samples.

Run No	Fiber Content (wt.%)	Fiber Size (μm)	Chemical Treatments	Flexural Frequency $\left(f_{f})-[\mathrm{Hz}\right]$	Torsional Frequency $\left(f_{t})-[\mathrm{Hz}\right]$	Young’s Modulus (E)-[MPa]	Shear Modulus (G)-[MPa]
1	6	150	3	28.8	13.9	10.5	3.8
2	2	150	1	36.7	17.7	17.2	6.2
3	4	150	2	27.2	13.1	9.5	3.4
4	2	75	2	29.0	14.0	10.6	3.9
5	6	225	2	26.2	12.7	8.6	3.1
6	4	225	1	34.9	16.9	15.6	5.7
7	4	225	3	26.1	12.6	8.8	3.2
8	4	150	2	27.8	13.4	9.7	3.5
9	4	75	1	29.4	14.2	10.9	3.9
10	4	75	3	34.3	16.6	15.0	5.5
11	2	150	3	27.6	13.3	9.7	3.5
12	6	150	1	29.9	14.4	11.5	4.2
13	4	150	2	27.9	13.5	9.8	3.6
14	6	75	2	29.6	14.3	11.3	4.1
15	2	225	2	28.6	13.8	10.4	3.8

**Table 4:** Comparison between experimental and predicted values for elastic constants.

Run	Young’s Modulus (MPa)			Shear Modulus (MPa)		
	Experimental	Predicted	Error %	Experimental	predicted	Error %
1	10.5	11.7	10.1	3.8	4.2	9.8
2	17.2	16.0	7.4	6.2	5.8	7.5
3	9.5	9.7	2.1	3.4	3.5	2.1
4	10.6	10.9	2.6	3.9	4.0	2.4
5	8.6	8.3	3.4	3.1	3.0	3.9
6	15.6	16.2	3.4	5.7	5.8	3.1
7	8.8	7.9	11.4	3.2	2.8	11.8
8	9.7	9.7	0.5	3.5	3.5	0.9
9	10.9	11.8	7.6	3.9	4.3	7.4
10	15.0	14.5	3.9	5.5	5.2	3.8
11	9.7	9.9	2.5	3.5	3.6	2.3
12	11.5	11.3	2.3	4.2	4.1	2.6
13	9.8	9.7	1.7	3.6	3.5	2.0
14	11.3	10.7	5.9	4.1	3.8	6.0
15	10.4	11.0	5.7	3.8	4.0	5.3

**Table 5:** ANOVA results for Young’s modulus.

Source	DF	Adj SS	Adj MS	F-Value	*p*-Value
Model	9	86.6716	9.6302	7.63	0.019
Linear	3	22.4252	7.4751	5.92	0.042
A	1	4.4175	4.4175	3.50	0.120
B	1	2.4581	2.4581	1.95	0.222
C	1	15.5497	15.5497	12.32	0.017
Square	3	22.2585	7.4195	5.88	0.043
A×A	1	0.0395	0.0395	0.03	0.867
B×B	1	0.7613	0.7613	0.60	0.472
C×C	1	21.9549	21.9549	17.40	0.009
2-Way Interaction	3	41.9879	13.9960	11.09	0.012
A×B	1	1.4591	1.4591	1.16	0.331
A×C	1	10.4499	10.4499	8.28	0.035
B×C	1	30.0790	30.0790	23.84	0.005
Error	5	6.3088	1.2618		
Lack-of-Fit	3	6.2332	2.0777	54.98	0.018
Pure Error	2	0.0756	0.0378		
Total	14	92.9804			

**Table 6:** ANOVA results for shear modulus.

Source	DF	Adj SS	Adj MS	F-Value	*p*-Value
Model	9	11.5319	1.28133	7.79	0.018
Linear	3	3.0062	1.00207	6.09	0.040
A	1	0.5864	0.58639	3.57	0.118
B	1	0.3313	0.33126	2.01	0.215
C	1	2.0886	2.08857	12.70	0.016
Square	3	2.9324	0.97745	5.94	0.042
A×A	1	0.0036	0.00361	0.02	0.888
B×B	1	0.0990	0.09901	0.60	0.473
C×C	1	2.8912	2.89125	17.58	0.009
2-Way Interaction	3	5.5934	1.86445	11.34	0.011
A×B	1	0.1911	0.19114	1.16	0.330
A×C	1	1.4078	1.40784	8.56	0.033
B×C	1	3.9944	3.99437	24.29	0.004
Error	5	0.8224	0.16447		
Lack-of-Fit	3	0.8105	0.27016	45.52	0.022
Pure Error	2	0.0119	0.00594		
Total	14	12.3543			

**Table 7:** Optimized combination of parameters.

S.No	Fiber Content (wt.%)	Fiber Size (μm)	Chemical Treatment	Elastic Constants	Experimentation	Optimization	Error (%)
1	2	225	1	Young’s Modulus (MPa)	18.2	19.2	5.1
2	2	225	1	Shear Modulus (MPa)	6.6	7.0	5.7

## Data Availability

Not applicable.
